# Complete Genome Sequence of Staphylococcus epidermidis CSF41498

**DOI:** 10.1128/MRA.01138-18

**Published:** 2019-01-10

**Authors:** M. R. Galac, Tra-My Hoang, Ana C. Ong, M. Hinkle, J. P. O’Gara, P. D. Fey

**Affiliations:** a Multidrug-Resistant Organism Repository and Surveillance Network (MRSN), Walter Reed Army Institute of Research, Silver Spring, Maryland, USA; b Department of Pathology and Microbiology, University of Nebraska Medical Center, Omaha, Nebraska, USA; c Department of Microbiology, School of Natural Sciences, National University of Ireland, Galway, Ireland; Indiana University, Bloomington

## Abstract

Staphylococcus epidermidis CSF41498 is amenable to genetic manipulation and has been used to study mechanisms of biofilm formation. We report here the whole-genome sequence of this strain, which contains 2,427 protein-coding genes and 82 RNAs within its 2,481,008-bp-long genome, as well as three plasmids.

## ANNOUNCEMENT

Staphylococcus epidermidis is a commensal bacterium and opportunistic pathogen that generally colonizes the human skin and mucous membranes (1, 2). Because of its prevalence, S. epidermidis is one of the major causes of nosocomial infections due to its ability to form biofilms on medical implant devices (3). These biofilm infections are extremely difficult to treat, owing to their inherent resistance to the host immune system and antibiotics (4–7).

CSF41498 is a biofilm-forming strain originally isolated from cerebrospinal fluid in Dublin, Ireland (8). This strain is susceptible to several antibiotics, including erythromycin, trimethoprim, tetracycline, kanamycin, and chloramphenicol, facilitating the introduction of plasmids and generation of marked mutations. Furthermore, genetic manipulation of this strain is possible due to the ability to move DNA via electroporation and transduction using bacteriophages ΦA6C and Φ187 (9, 10). In addition, CSF41498 has been used to study multiple aspects of biofilm formation, making this strain and its genome sequence useful tools (8, 11–16).

For DNA extraction, S. epidermidis CSF41498 was grown on blood agar plates overnight at 37°C. DNA was then extracted using the DNeasy UltraClean microbial kit (Qiagen, Germany) per the manufacturer’s directions. Sequencing was performed as previously described (17). RS II (Pacific Biosciences, USA) single-molecule real-time (SMRT) sequencing produced 116,695 reads, with an average length of 14,633 bp. The reads were assembled using HGAP2 in the SMRT Analysis portal into three polished contigs representing one chromosome and two plasmids. MiSeq (Illumina, Inc., USA) short-read sequencing produced 1,526,588 paired-end reads with an average length of 300 bp and insert size of 500 bp. These reads were mapped to the trimmed and circularized SMRT sequences using the mapper within Geneious (Biomatters, New Zealand) to correct the errors in the long-read sequencing, resulting in an average depth of coverage of 89× for the chromosome and 150× for the plasmids. Reads that did not map to any of the long-read contigs were assembled by the Geneious *de novo* assembler to form a third plasmid around 8 kb in length, with an average read depth of 850×. This plasmid is smaller than the fragmentation size for the long-read sequencing, which is why there was no contig detected from the SMRT assembly; however, as verification, we found long reads that did map to the plasmid. Genes were predicted using the NCBI Prokaryotic Genome Annotation Pipeline version 4.5 (18).

The genome of strain CSF41498 is 2,481,008 bp long, containing 2,427 protein-coding sequences (CDSs) and 82 RNAs, with 32.2% GC content. The plasmid pCSF41498_1 comprises 24,519 bp coding for 26 CDSs. The plasmid pCSF41498_2 comprises 21,583 bp coding for 27 CDSs. The plasmid pCSF41498_3 comprises 8,256 bp coding for 8 CDSs.

### Data availability.

The complete genome sequence of S. epidermidis CSF41498 has been deposited in GenBank under the accession numbers CP030246 to CP030249. The sequencing reads have also been deposited in GenBank under accession number SRS3935761.
